# On the Importance of Separation and Labelling on the Hypersphere †

**DOI:** 10.3390/e28080870

**Published:** 2026-08-01

**Authors:** Martin Lindström, Ragnar Thobaben, Mikael Skoglund

**Affiliations:** Department of Information Science and Engineering, KTH Royal Institute of Technology, 100 44 Stockholm, Sweden; ragnart@kth.se (R.T.); skoglund@kth.se (M.S.)

**Keywords:** hyperspherical clustering, representation geometry, cluster separation, cluster labelling, prototypical learning

## Abstract

Good representations strive for diversity among dissimilar features, often by mapping latent representations onto the hypersphere and enforcing separation. At the same time, the latent space structure should align with data semantics. While prior work propagates the need for separation, it is difficult to systematically analyse and determine the importance that feature cluster separation and semantic alignment have on downstream performance. In this paper, we address this gap in the understanding of hyperspherical clustering methods and provide new insights into which properties promote good performance. Firstly, we introduce a principled analysis framework which disentangles the importance of cluster separation and labelling on performance. Secondly, we give a full characterisation of the optimal cluster separation, both through theoretical analysis and practical schemes that achieve near-optimal separation. The results show that even though cluster separation contributes to performance, especially in low dimensions, performance gains obtained by matching the cluster labelling to the input data structure are more significant, and aligning cluster labelling with the underlying data structure can compensate for poor separation.

## 1. Introduction

Representation learning aims at learning low-dimensional representations of data which retain useful information and are easy to use in downstream tasks. Geometric structure-inducing inductive biases have long been popular in representation learning. Two common such biases are norm-invariant latent representations, either through explicit normalisation to the hypersphere or scale-invariant loss functions, and representation separation, which ensures diverse representations.

Normalising representations to the hypersphere has seen empirical success in a wide range of settings: variational autoencoders [1,2], self-supervised learning [3,4,5,6,7,8], and supervised learning [9,10,11,12,13,14,15,16,17,18,19,20]. It has been shown empirically to improve quality [6] and theoretically stabilise training behaviour when using SoftMax functions [4]. Moreover, normalisation makes geometric separation a meaningful quantity for linear separability, which is a common quality metric for representations [3]. Cluster separation has been motivated as an inductive bias to avoid collapse in self-supervised settings [21] and as an intuitive objective in supervised settings [18,19,20].

Across these settings, it is hard to quantify the importance of separation on linear separability. One may impose a geometric constraint on a set of clustering points, thereby aiming for separation, and then align the semantic structure of the data with this geometry. Together, these actions may result in tight and well-separated clusters which are linearly separable. However, it is hard to quantify how much of downstream performance is explained by separation compared to the semantic structure of the latent space. In particular, many common objective functions (including information-theoretic objectives) are invariant to cluster relabelling and many other geometry-altering transformations. Therefore, different latent geometries may not be easy to realise with typical training algorithms.

In this paper, we systematically analyse cluster separation and the semantic structure of the latent space, as well as their impact on classification performance. We show that optimising cluster labelling yields larger performance gains and can, in many cases, compensate for poor cluster separation. In contrast, separation is most consequential in low-dimensional settings where the geometry is more constrained. As an analysis framework to separate these effects, we use hyperspherical prototypical learning, which allows us to control cluster separation and labelling independently and quantify their effect on linear separability. Specifically, our contributions are as follows:

(i) We introduce an analysis framework with fixed cluster prototypes and trainable labelling. This enables separation and labelling to be varied and quantified independently.
(ii) We analyse and optimise hyperspherical separation. Using theory and algorithms from error-correcting codes, we present both sharp bounds on the optimal cluster separation, as well as efficient algorithms that achieve near-optimal separation in low dimensions. This allows us to systematically compare near-optimal and poor separation schemes.
(iii) Our performance evaluation on the CIFAR-100 and ImageNet benchmarks reveals that poor cluster separation can often be compensated by good cluster labelling. Near-optimal prototype separation has a significantly smaller impact on performance than anticipated, and it is most important in low dimensions where degrees of freedom are scarce.

We note that Contribution (ii) is based on our preliminary work [22], and the remaining contributions add significant context and extension to this work.

The remainder of this paper is organised as follows: Related works and discussions are given in Section 2. We present our analysis framework in Section 3 and give theoretical justifications in Section 4. The analysis of optimal cluster separation is given in Section 5, and performance evaluations are discussed in Section 6. Section 7 concludes this paper.

## 2. Background

Many supervised and self-supervised methods aim to induce tight and well-separated hyperspherical clusters. We are concerned with analysing the roles of cluster separation and labelling in hyperspherical learning. Throughout this paper, we use the term “prototypes” to refer to hyperspherical elements used to structure the latent space; these could be codebook entries in a vector quantiser, trainable cluster centres or averaged representations in self-supervised learning, fixed cluster centres, or similar. Separation then means to impose geometric inductive biases on prototypes, and labelling refers to how semantic structures like classes or pseudo-labels are mapped onto this geometry. We utilise hyperspherical prototypical learning, a supervised learning method, as an analysis framework in which we can vary separation and labelling *independently* while connecting both to a concrete performance metric related to clustering linear separability in terms of accuracy.

As motivating examples, consider the toy example and pre-trained self-supervised models in Appendix A. They suggest that cluster separation is neither necessary nor sufficient for performance and that cluster labelling may be a more important factor. Moreover, we give a counter-example where poor separation increases performance since we match the geometry of the latent space to the data. These observations motivate careful study of the relationship between separation, labelling, and performance.

In the following, we start with a review of important hyperspherical clustering methods and the need to optimise cluster labelling. We then formalise hyperspherical prototypical learning as a widely applicable analysis framework and review important results on hyperspherical separation.

### 2.1. Clustering-Based Methods

Hyperspherical clustering is an implicit or explicit ingredient in several representation learning paradigms. In contrastive and multi-view self-supervised learning, objectives encourage alignment of related samples and uniformity of representations, which together induce clustering behaviour [3,6]. A related information-theoretic analysis has connected many multi-view self-supervised methods to reconstruction and entropy, which are related to clustering and spread, respectively [21].

Several methods explicitly introduce prototypes (sometimes called centroids, code vectors, or codebooks) and learn assignments with respect to them. This includes prototype-based self-supervised learning and vector quantisation-based methods [8,23,24,25]. In supervised learning, closely related methods arise when class-based prototypes are used [9,10,11,12,13,14,15,16,17,18,19,20,22]. These classes of methods motivate a careful separation of factors which are hard to disentangle in practice: (i) the geometric inductive bias on prototype separation and (ii) the semantic structure of the latent space through labelling.

We note that prototype-based methods are not the only way to induce well-separated and semantically meaningful clusters, and to the best of our knowledge, it is still an open problem with respect to how best to achieve these aims. A broader analysis of different geometric constraints are beyond the scope of this paper.

### 2.2. Connecting Prototype Separation to Linear Separability

Tight, well-separated hyperspherical clusters are a sufficient condition for linear separability, but prototype separation alone does not explain downstream performance in realistic settings. For example, a classical theoretical result for support vector machines shows that tight and well-separated clusters generalise well ([26], Theorem 15.4). However, this result is agnostic to how easy or hard the latent geometry is to realise with a given backbone and training algorithm.

Recent theoretical works in representation learning instead indicate that the simplicity of the latent space geometry is important. In [27], a bound on the generalisation error of supervised representation learning was derived, where simpler latent variables are shown to generalise better. Their bound is inspired by theoretical analyses of the information bottleneck method, where experimental evidence indicates that geometric simplicity is important in characterising performance. Specifically, ref. [28] empirically showed that a simple latent space geometry can be important in both generalisation and robustness to noisy inputs.

Recent empirical works have argued similarly. The relabelling of prototypes was shown to be important in hyperspherical prototypical learning in [18], where the authors matched the distance between the word embeddings of the class names with the induced prototype separation. The authors showed that this could substantially increase classification accuracy, particularly in low latent space dimensions. A variable class-to-prototype mapping was proposed in [20] which was used on top of the fixed prototypes proposed in [18]. In our preliminary work [22], we showed that there was a correlation between induced separation and separability but not causation: Some mutually well-separated prototype sets yielded poor linear separability and vice versa. These observations align with the motivating examples in Appendix A: Two settings can have comparable prototype separation yet differ substantially in downstream performance depending on how the induced latent geometry aligns with data semantics, backbones, and optimisers.

### 2.3. Supervised Hyperspherical Prototypical Learning as an Analysis Framework

In this paper, we use hyperspherical prototypical learning, a supervised learning method, to analyse hyperspherical clustering-based methods more generally. For this, we require a method where (i) there is a clear connection between clustering and performance, and (ii) it is possible to disentangle clusters from other geometrical considerations. Hyperspherical prototypical learning fulfils both these requirements: Knowing the number of classes allows us to pick the right number of prototypes and unambiguously tie clustering to accuracy; fixed prototypes allow us to analytically find the optimal separation and empirically connect it to performance. Self-supervised methods and methods with trainable prototypes are hard to analyse and are therefore not considered further. For requirement (i), self-supervised methods have two major shortcomings: It is unclear how many clusters are needed, and it is hard to connect clustering based on self-similarity to a performance metric like classification accuracy. For requirement (ii), we require clusters which are fixed prior to training. Hence, we retain the clustering desiderata from self-supervised methods and methods with trainable prototypes in our analysis framework, but we impose additional restrictions for a tractable analysis.

Finally, we briefly justify our restriction to separation on the hypersphere. In an unbounded space, it is always possible to increase class *separation* without making classes more *(linearly) separable*. For example, we can always increase separation between prototypes $c_{i}$ and $c_{j}$ by a factor *k* through scaling, $∥kc_{i}-kc_{j}∥=k∥c_{i}-c_{j}∥$, which does not, however, improve separability. Hence, separation is meaningful in a hyperball. Moreover, the restriction to the hypersphere is only a small relaxation: In high dimensions, most of the volume of a hyperball is concentrated around the surface.

### 2.4. Theoretical Background on Hyperspherical Separation

#### 2.4.1. The Sphere-Packing Problem

The problem of placing $N_{C}$ maximally separated points on the *n*-dimensional unit hypersphere $S^{n-1}$ is well studied [29]. Sometimes called the Tammes problem [30] or the sphere-packing problem, it can be formulated mathematically through the non-convex and combinatorial optimisation problem:

(1) $$\begin{array}{c} \min_{C}\max_{i\neq j}\;\langle c_{i},c_{j}\rangle , \end{array}$$

where $C:=\{c_{i}\in S^{n-1}:i=1,\ldots ,N_{C}\}$ is a set of $N_{C}$ points, or prototypes, on the hypersphere. This problem can be equivalently formulated as maximising the minimum Euclidean distance $∥c_{i}-c_{j}{∥}^{2}=2-2\langle c_{i},c_{j}\rangle$ for i≠j since $∥c_{i}∥=1$ on the hypersphere, or equivalently, it can be formulated as minimising the maximum cosine similarity $\cos\alpha_{ij}=\langle c_{i},c_{j}\rangle$ for i≠j again since $∥c_{i}∥=1$. Here, we find the formulation in (1) to be the most mathematically convenient. Unfortunately, the problem is unsolved for arbitrary numbers of prototypes $N_{C}$ in a given dimension *n* [29], and it is even unsolved for general $N_{C}$ in dimension n=3 [31]. However, we emphasise that problem (1) is exactly the problem of maximising prototype separation on the hypersphere.

#### 2.4.2. Approximate Solutions

Several approximate solutions to (1) have been proposed. In Mettes et al. [18], a projected gradient descent scheme was employed to tackle non-convexity, and relaxation to the combinatorial constraint in i≠j was proposed. However, the authors did not show if this scheme yielded solutions that are close to optimal. Later, Kasarla et al. [19] proposed relaxation to the worst-case separation problem, where the mutual separation between prototypes was restricted to being constant, $\langle c_{i},c_{j}\rangle =\mathrm{const}.$, and provided a closed-form solution in dimension $n=N_{Y}-1$. Likewise, the authors did not show whether their solutions are near-optimal. We address these limitations by providing a full characterisation of the solutions to (1) and practical schemes that are provably near-optimal in low dimensions Section 5. We also analyse the schemes from Mettes et al. [18], Kasarla et al. [19] in light of this characterisation.

### 2.5. Open Questions

In summary, related works show that representation separation on the hypersphere is a natural and hard problem to solve both in supervised and self-supervised settings. There are a plethora of schemes to induce separation, as well as justifications as to why it is important. However, there are both theoretical and empirical results that indicate that inducing separation is *not enough*: they indicate that aligning the latent space geometry to the input data structure is important.

Previous works often fail to separate the effects of cluster separation and labelling on downstream performance. We bridge the gap in previous works by systematically disentangling these factors and their effect on performance. In particular, we (i) propose a framework which decouples prototype geometry from semantic labelling, which enables us to disentangle their impact on performance; (ii) provide a tight characterisation of achievable hyperspherical separation; and (iii) perform performance evaluations.

## 3. Method

We now present the learning system that we use to systematically disentangle the impact of cluster separation and labelling on performance. Our method follows the standard hyperspherical prototypical learning framework, with an additional trainable relabelling.

### 3.1. Framework and Learning Objective

**Figure 1:**
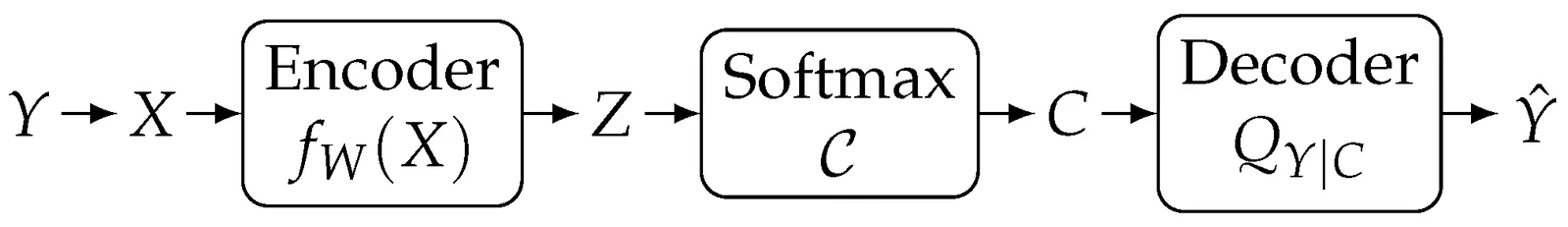
System model.

Consider the system shown in Figure 1 and assume we have a classification task with the data generating model $P_{X,Y}=P_{X|Y}P_{Y}$, where $P_{Y}$ is known. Assume that we are given a dataset $S_{m}={\left\{\left(x_{i},y_{i}\right)\right\}}_{i=1}^{m}$ where $x_{i}\in X$ belongs to class $y_{i}\in \left\{1,\ldots ,N_{Y}\right\}$. Consider a combined clustering and classification system where prototypes constitute pre-defined cluster centres on the hypersphere $C:=\{c_{i}\in S^{n-1}:i=1,\ldots ,N_{C}\}$. These prototypes are specified before training by an approximate solution to (1) and held fixed. A sample *X* is first mapped to the hypersphere using $Z=f_{W}\left(X\right)$ and then assigned to cluster *C* with probability vector

(2) $$Q_{C|Z}=\mathrm{softmax}\left(Z,C\right)={\left[\frac{\exp(\langle Z,c_{i}\rangle /\tau )}{\sum_{j}\exp\left(\langle Z,c_{j}\rangle /\tau \right)}\right]}_{i=1}^{N_{C}},$$

where τ is a temperature parameter.

To improve the ability of hyperspherical prototypical learning to preserve some data structure, we also introduce a trainable relabelling of prototype $Q_{Y|C}$. Equivalently, this can be seen as a trainable mapping between clusters and classes. In this way, we induce separation in the latent space by the choice of prototypes while still leaving flexibility to preserve structure in the input data. We note that this framework differs from the standard prototypical learning setting in [18,19,22], which uses a fixed mapping from classes to prototypes. With some abuse of notation, we use $Q_{Y|C}$ both as a matrix ($N_{Y}\times N_{C}$ with elements $Q_{Y=y|C=i}$) and as a Markov kernel (denoting the transition probability from *C* to *Y*). Using the matrix notation, we have $Q_{Y|Z}=Q_{Y|C}\;Q_{C|Z}$. We consider the case where $N_{Y}=N_{C}$ to isolate the effects of separation and labelling: Introducing multiple prototypes per class introduces additional degrees of freedom that may obscure the causal relationship between prototype separation and performance.

Clearly, we would like cluster relabelling, which assigns *one* cluster to *one* class; equivalently, $Q_{Y|C}$ should be a permutation matrix. Since permutation matrices are sparse doubly stochastic matrices, we propose the following loss function:

(3) $$L\left(x,y;W,Q_{Y|C}\right)=\ell_{\mathrm{CE}}\left(P_{Y=y|x},Q_{Y=y|x};W,Q_{Y|C}\right)+\lambda \ell_{\mathrm{sparse}}\left(Q_{Y|C}\right),$$

where $\ell_{\mathrm{CE}}$ is the typical cross-entropy loss; we emphasise the dependence on both the backbone parametrised by *W* and the class relabelling $Q_{Y|C}$, and $\ell_{\mathrm{sparse}}$ is a sparsity penalty on $Q_{Y|C}$. We choose to train both *W* and $Q_{Y|C}$ in an end-to-end manner using backpropagation for simplicity. For an information-theoretic motivation of the learning objective, we refer to Section 4.

### 3.2. Maximising Prototype Separation

Two approaches to maximising prototype separation are considered, coding-theoretic prototypes and optimisation-based prototypes. Here, we outline the overall approach and delegate the detailed analysis to Section 5.

Coding-theoretic prototypes rely on concepts from binary error-correcting codes to map *n*-dimensional binary vectors to the *n*-dimensional hypersphere $S^{n-1}$. This is a relaxation to the original problem (1), which admits a tight characterisation of achievable and optimal cluster separation: it turns out that coding-theoretic prototypes have *provably near-optimal separation in low dimensions*.

Optimisation-based prototypes propose relaxations to the original problem (1), which is needed because it is non-convex and combinatorial. We propose convex relaxation, which we show empirically is optimal in many settings. We also analyse schemes from the literature, particularly those proposed in [18,19], and we show that the relaxation in Mettes et al. [18] is *suboptimal* while the relaxation in Kasarla et al. [19] is *optimal* but inflexible.

### 3.3. Learning a Relabelling

We now turn to the problem of finding a suitable sparsity penalty $\ell_{\mathrm{sparse}}$ in (3). Starting from an arbitrary matrix M, a candidate $Q_{Y|C}$ can be created as follows: We apply a rectifying function element-wise to ensure that $m_{ij}>0$, after which we run Sinkhorn’s algorithm [32], which takes a strictly positive matrix, alternately normalises over rows and columns, and yields a doubly stochastic matrix. After this, a sparsity penalty may be applied through gradient descent, and in this way, we can iteratively refine candidate relabelling.

There are many sparsity penalties that provably yield sparse matrices. We would like this penalty to be fast to compute and track the number of non-zero elements well. Comparing penalties using the Frobenius norm, entropy, and AutoShuffle loss [33], we find that the AutoShuffle loss is most suitable. We defer the comparison to Appendix B. The AutoShuffle loss is defined by

(4) $$\ell_{\mathrm{AutoShuffle}}\left(M\right)=\sum_{i=1}^{n}\left[\sum_{j=1}^{n}\left|m_{ij}\right|-{\left(\sum_{j=1}^{n}m_{ij}^{2}\right)}^{1/2}\right]+\sum_{j=1}^{n}\left[\sum_{i=1}^{n}\left|m_{ij}\right|-{\left(\sum_{i=1}^{n}m_{ij}^{2}\right)}^{1/2}\right].$$

## 4. Theoretical Motivation

In this section, we use an information-theoretic lens to show that our framework allows us to disentangle the importance of separation and labelling. Our argument is presented in three steps. Firstly, we show that large mutual information between the representations and labels is necessary for good accuracy and that standard cross-entropy training encourages this. Secondly, however, mutual information is invariant to invertible transformations in the representation space, particularly to cluster relabellings. Therefore, even if a training algorithm reaches the global optimum, the cross-entropy objective does not optimise latent space geometry. Finally, we note that the proposed framework allows us to optimise labelling separately; in other words, it allows us to disentangle the impact of cluster labelling on performance.

### 4.1. Analysis

To formalise our analysis framework in Figure 1, we have the Markov chain $Y-X-Z-\hat{Y}$, with the data distribution $P_{X,Y}=P_{X|Y}P_{Y}$. Our aim is to guess *Y* from *X*, but since $P_{Y|X}$ is intractable, we instead train a deterministic mapping $Z=f_{W}\left(X\right)$ and guess $\hat{Y}$ from $Q_{Y|Z}=Q_{Y|C}Q_{C|Z}$.

We now show that large mutual information between *Z* and *Y* is *necessary* for good performance and that the standard cross-entropy loss function maximises a lower bound on this mutual information.

**Proposition** **1.** *Assume that Y is uniformly distributed over $\left\{1,\ldots ,N_{Y}\right\}$, and let $P_{e}$ denote the error probability $P_{e}:=P\left\{Y\neq \hat{Y}\right\}$. Then,*

(5) $$P_{e}\geq 1-\frac{I(Y;Z)+\log2}{\log N_{Y}}.$$

**Proof.**  By Fano’s inequality ([34], Theorem 3.12), the conditional entropy H(Y∣Z) is related to $P_{e}$ through

(6) $$H\left(Y|Z\right)\leq P_{e}\log\left(N_{Y}-1\right)+H_{b}\left(P_{e}\right)\leq P_{e}\log N_{Y}+\log2,$$

where $H_{b}\left(x\right)=-x\log x-\left(1-x\right)\log\left(1-x\right)$ is the binary entropy function, which is bounded by $H_{b}\left(x\right)\leq \log2$. Now, assuming that *Y* is uniformly distributed, we obtain a lower bound on mutual information through

(7) $$I\left(Y;Z\right)=H\left(Y\right)-H\left(Y|Z\right)\geq \log N_{Y}-P_{e}\log N_{Y}-\log2.$$

Solving this for $P_{e}$ yields the claim. □

Note that similar analyses can be done both in the cases of non-uniform distributions over *Y* and if $N_{Y}$ is unbounded; for details, see ([34], Theorem 3.12). Also, we note that this extends to *any* intermediate processing step; retaining large mutual information is necessary for good classification accuracy.

Next, we show that minimising cross-entropy pushes $f_{W}$ to preserve the mutual information I(Y;Z):

**Proposition** **2.** *Negative cross-entropy, together with label entropy, provides a lower bound on mutual information through*

(8) $$I\left(Y;Z\right)\geq E_{P_{X,Y}}[\log Q_{Y|X}]+H\left(Y\right).$$

**Proof.**  We have

(9) $$\begin{array}{cc} I(Y;Z) & =E_{P_{Y,Z}}\left[\log\frac{P_{Y|Z}}{P_{Y}}\right]=E_{P_{Z,Y}}\left[\log\frac{P_{Y|Z}}{P_{Y}}\frac{Q_{Y|Z}}{Q_{Y|Z}}\right] \end{array}$$

(10) $$\begin{array}{cc} \; & =E_{P_{Y,Z}}\left[\log Q_{Y|Z}\right]+H\left(Y\right)+E_{P_{Y,Z}}\left[\log\frac{P_{Y|Z}}{Q_{Y|Z}}\right], \end{array}$$

where the last term is conditional relative entropy $E_{P_{Y,Z}}\left[\log\frac{P_{Y|Z}}{Q_{Y|Z}}\right]=D(P_{Y|Z}∥Q_{Y|Z}\left|P_{Z}\right)$, which is non-negative. Finally, notice that since $f_{W}$ is deterministic, we have $P_{Z|X}\left(z|x\right)=\delta_{f_{W}\left(x\right)}\left(z\right)$, which yields $E_{P_{Y,Z}}\left[\log Q_{Y|Z}\right]=E_{P_{Y,X}}\left[\log Q_{Y|Z=f_{W}\left(X\right)}\right]=E_{P_{Y,X}}\left[\log Q_{Y|X}\right]$. □

To summarise, we have shown that minimising cross-entropy maximises a lower bound on mutual information, which is necessary for good accuracy. However, both I(X;Y) and I(Z;Y) are invariant to the location and ordering of clusters ([34], Theorem 1.4(c)); hence, cross-entropy loss does not optimise latent space geometry. This allows us to optimise geometry separately in (3), thereby allowing us to investigate the impact of labelling on performance.

### 4.2. Discussion

We now discuss some implications of our analysis.

Firstly, we discuss where information is lost in this framework. By the data processing inequality, we have $I\left(X;Y\right)\geq I\left(Z;Y\right)\geq I\left(\hat{Y};Y\right)$. However, note that if $Q_{Y|C}$ is a permutation matrix, it is also information-preserving. Moreover, if *Z* is a sufficient statistic of *X* for *Y* ([34], Theorem 3.9(d)), then (8) reaches its maximum I(X;Y)=I(Z;Y). Hence, the proposed framework strives to minimise the information loss by simultaneously aligning *Z* with prototype C via $f_{W}$ and solving the labelling problem via the sparsity loss of the Markov kernel $Q_{Y|C}$.

Secondly, this framework aims to provide a lower bound on mutual information with downstream performance. Tschannen et al. [35] showed empirically that tighter lower bounds on mutual information may not result in better downstream performance. Moreover, even if a representation has provably high mutual information, its downstream performance need not be good. These issues are overcome in our framework: we optimise performance over a parameter which mutual information is invariant to.

Thirdly, in this context, information-theoretic motivations are not inherently vacuous. McAllester and Stratos [36] showed that high-confidence lower bounds on mutual information based on *m* samples cannot be larger than O(logm), potentially leaving a large gap. In a supervised setting, this result is not inherently problematic. Since $I\left(Y;Z\right)\leq \log N_{Y}$, lower bounds on mutual information can indeed reach the optimum given that $N_{Y}$ is (sufficiently) smaller than *m*.

Our main takeaways are as follows. High mutual information I(Y;Z) is necessary for high accuracy, and cross-entropy loss is a bound on this quantity. However, mutual information is invariant to invertible transformations in the representation space, particularly to cluster relabelling. This, therefore, motivates separate relabelling optimisation in order to determine which factors impact performance.

## 5. Analysing and Inducing Hyperspherical Separation

In this section, we give a full characterisation of the optimal hyperspherical separation (see Theorem 1), as well as practical schemes which are optimal or near-optimal. In particular, we use theoretical tools from linear binary error-correcting codes to obtain an *achievability bound*: There are configurations that are no worse than orthogonal prototypes in low dimensions. Using tools from spherical coding theory, we then give a *converse bound*: Minimum separation cannot be improved beyond near-orthogonality. The analysis and practical schemes are used in Section 6 to connect separation to accuracy.

To provide some intuition about coding-theoretic prototypes, consider a mapping of *n*-dimensional binary vectors to the *n*-dimensional hypersphere $S^{n-1}$ through $\gamma :{\left\{0,1\right\}}^{n}\to S^{n-1}$, defined by

**Figure 2:**
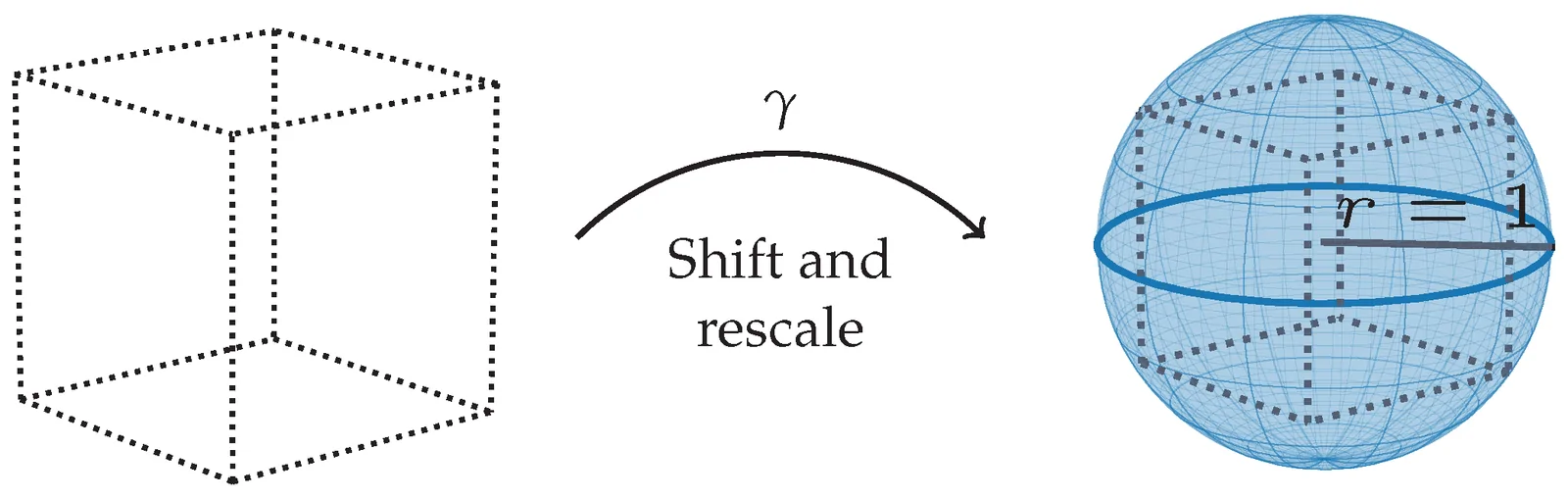
How coding-theoretic prototypes are constructed. We map the *n*-dimensional Hamming cube (**left**) via the mapping γ defined in (11) (**Centre**) to the unit hypersphere (**right**).

(11) c=γ(b):=2(b−12)n,

for any binary vector b. For an illustration, see Figure 2. Through this mapping, we have ∥c∥=1, and all components of c lie on c(k)∈{−1n,+1n}. Since there are $2^{n}$ binary vectors of length *n*, this allows us to place $2^{n}$ distinct points on the hypersphere, each with a cosine similarity of at most 〈c,c′〉≤1−2n for c=γ(b) and $c^{\prime }=\gamma \left(b^{\prime }\right)$ and distinct binary vectors $b\neq b^{\prime }$. The key insight lies in the fact that we are only interested in finding $N_{C}<2^{n}$ prototypes, typically *far* fewer prototypes than $2^{n}$. Hence, by selecting a binary codebook $B:=\{b_{i}\in {\left\{0,1\right\}}^{n}:i=1,\ldots ,N_{C}\}$ of well-separated binary vectors, we can *guarantee* well-separated prototypes. In fact, for each differing bit in b and $b^{\prime }$, we gain an additional separation of 2n in terms of cosine similarity.

### 5.1. Background on Binary Block Codes

We begin by giving a brief background on coding theory. The interested reader is referred to MacWilliams and Sloane [37] for a complete treatment. A binary block code with parameters [n,k] consists of a codebook B of $2^{k}$ binary codewords of length *n*, as well as a bijective encoder that maps all $2^{k}$ binary vectors of length *k* to a codeword. The encoding adds n−k bits of redundancy which can later be used to detect and correct errors. Moreover, the *rate* of the code is defined as R=kn, which captures the redundancy added: A low rate implies high redundancy and vice versa.

The error detection and correction capability of B relies on the *separation* between codewords $b_{i},b_{j}\in B$ in terms of the Hamming distance, namely

(12) $$d_{H}\left(b_{i},b_{j}\right):=\sum_{\ell =1}^{n}I\left\{b_{i}\left(\ell \right)\neq b_{j}\left(\ell \right)\right\}.$$

Moreover, a binary code with the minimum Hamming distance

(13) $$d_{\min}:=\min_{b_{i},b_{j}\in B,i\neq j}d_{H}\left(b_{i},b_{j}\right)$$

can *detect* $d_{\min}-1$ errors and *correct*⌊(dmin−1)2⌋ errors.

In our setting, we fix $k=\lceil \log_{2}N_{Y}\rceil$, which is typically in the high-redundancy regime. In this regime, we expect error-correcting codes to offer large separation in terms of the minimum Hamming distance. A fundamental result in this direction is that *good codes exist*, and they are formalised by the well-known Gilbert–Varshamov bound ([37], Ch. 1, Thm. 12).

**Lemma** **1** (Gilbert–Varshamov Bound). *There exists an [n,k] code with a minimum distance of at least $d_{\min}$ provided that*

(14) 2n−k>∑i=0dmin−2n−1i.

The Gilbert–Varshamov bound guarantees that *there is* a code with a minimum distance of at least $d_{\min}$, but it does not inform us of *how to find* one. Fortunately, as we will soon see, there exist several linear binary codes with better minimum distances.

Note that the bound in (14) needs to be evaluated carefully in order to avoid numerical issues. However, the bound can be rewritten as

(15) 2−k>∑i=0dmin−2n−1i12i12n−i=Fbindmin−2;n−1,12,

where $F_{\mathrm{bin}}$ denotes the cumulative density function of the binomial distribution, which is implemented in a numerically stable manner in many libraries.

### 5.2. Background on Spherical Coding

Hyperspherical prototype separation is studied in *spherical coding theory*, and it has been of interest since the 1950s when it was investigated by Shannon; it has been of continued interest since (see, for example, [29,38,39,40,41].)

Of importance to us is the Rankin bound, which provides a lower bound to prototype separation ([41], Theorem 1.4.1).

**Lemma** **2** (Rankin Bound). *Any set of $N_{C}$ hyperspherical prototypes C satisfies*

$$\min_{i\neq j}{∥c_{i}-c_{j}∥}^{2}\leq \frac{2N_{C}}{N_{C}-1}.$$

### 5.3. Theoretical Analysis

We now provide a formal proof that a well-separated binary codebook yields well-separated hyperspherical prototypes through γ in (11).

**Proposition** **3.** *Assume that B is the codebook of a binary [n,k] code with minimum distance $d_{\min}$. Then, for every pair $b_{i},b_{j}\in B$ with i≠j, the cosine similarity between $c_{i}=\gamma \left(b_{i}\right)$ and $c_{j}=\gamma \left(b_{j}\right)$ is upper bounded by*

(16) $$\langle c_{i},c_{j}\rangle =1-\frac{2d_{H}\left(b_{i},b_{j}\right)}{n}\leq 1-\frac{2d_{\min}}{n}.$$

**Proof.**  Notice that two binary vectors differing in $d_{H}\left(b_{i},b_{j}\right)$ positions obey $\sum_{\ell =1}^{n}{\left(b_{i}\left(\ell \right)-b_{j}\left(\ell \right)\right)}^{2}=d_{H}\left(b_{i},b_{j}\right)$. Expanding $∥c_{i}-c_{j}{∥}^{2}$ in two ways gives

(17) ∥ci−cj∥2=2−2〈ci,cj〉=∑ℓ=1n2(bi(ℓ)−12)−2(bj(ℓ)−12)n2.

Rearranging this expression and recalling that $d_{H}\left(b_{i},b_{j}\right)\geq d_{\min}$ yield the desired result. □

We are now ready to prove our main theoretical result on optimal prototype separation.

**Theorem** **1.** *There exists a set of hyperspherical prototypes* C *with cosine similarity at most (separation at least)*

(18) $$\max_{i\neq j}\;\langle c_{i},c_{j}\rangle \leq 1-\frac{2d_{\mathrm{GV}}}{n},$$

*where $d_{\mathrm{GV}}$ denotes the largest solution to the Gilbert–Varshamov bound in (14). Conversely, no set of prototypes exists with a maximum cosine similarity smaller (better separation) than*

(19) $$\max_{i\neq j}\;\langle c_{i},c_{j}\rangle \geq \frac{-1}{N_{C}-1}.$$

**Proof.**  The achievable bound (18) follows directly from combining Proposition 3 and Lemma 1. For the converse bound, recalling that $∥c_{i}-c_{j}{∥}^{2}=2-2\langle c_{i},c_{j}\rangle$, we apply Lemma 2 and carry out simplification, yielding (19). □

We make a few observations about these bounds. Firstly, we have the lower bound $\frac{-1}{N_{C}-1}\leq \min_{C}\max_{i\neq j}\langle c_{i},c_{j}\rangle$, and for moderate problem sizes, we have $\frac{-1}{N_{C}-1}\approx 0$. Moreover, the upper bound becomes tighter with *n* since $d_{\mathrm{GV}}$ grows with *n*: In fact, as we will soon demonstrate, we can find examples where $\max_{i\neq j}\langle c_{i},c_{j}\rangle =0$ in low-dimension n≈NC2. Our key conclusion is that our bounds are sharp: *configurations no worse than orthogonal prototypes are achievable*, and *minimum separation cannot be improved beyond near orthogonality*.

### 5.4. Practical Near-Optimal Schemes

We now give several examples of optimal and near-optimal prototypes, both using linear binary block codes and optimisation-based methods.

#### 5.4.1. Linear Binary Codes

We provide two examples of good codes: that is, where dmin=n2 in low-dimension $n<N_{Y}$. This implies that the separation is no worse than that of orthogonal prototypes. Moreover, these codes are easy to implement in Python 3 and therefore, they are easy to integrate into modern machine learning workflows. These codes are Reed–Muller (RM) and Bose–Chaudhuri–Hocquenghem (BCH) codes.

BCH codes are known to have good minimum distance properties in low-dimension $n<N_{Y}$ ([37], pp. 258). Although the exact minimum distance is not known analytically [42], we find empirically that it approaches dmin=n2 in dimensions $n<N_{Y}$. They are implemented in, for example, the Galois library ([43], v0.3.8).

The distance properties of RM codes are easier to analyse due to their simple construction [44]. In fact, they achieve separation no worse than that of orthogonal prototypes in dimension n≈NY2. We make this guarantee precise below.

**Lemma** **3** (Separation Guarantees for RM Codes). *Let $\tilde{N_{Y}}$ be the smallest power of 2 such that $\tilde{N_{Y}}\geq N_{Y}$. Then, RM codes in dimension n≥NY˜2 have a minimum distance of dmin=n2 and therefore guarantee that $\langle c_{i},c_{j}\rangle \leq 0$.*

**Proof.**  By construction, RM codes are [n,k] codes with $n=2^{m}$, k=∑i=0rmi, and a minimum distance of $d_{\min}=2^{m-r}$. Hence, we have cosine similarity $\langle c_{i},c_{j}\rangle \leq 0$ if r=1, namely if $1+m=1+\log_{2}n=k\geq \log_{2}\tilde{N_{Y}}\geq \log_{2}N_{Y}$ or if n≥NY˜2≥NY2. □

Linear binary codes provide a flexible way to derive prototypes, but there are restrictions on the dimensions that are realisable: RM codes are defined for $n=2^{m}$, and BCH codes are defined for $n=2^{m}-1$. Moreover, other dimensions are realisable with similar performance: Codes can be *punctured* by removing dimensions, thereby creating lower-dimensional prototypes, and they can be *extended* by adding dimensions, thereby creating higher-dimensional prototypes [37]. In general, puncturing a code can (but is not guaranteed to) reduce the minimum distance by 1, and extending a code can (but is not guaranteed to) increase the minimum distance by 1. Therefore, code-based prototypes have good hyperspherical separation around $n\approx 2^{m}$.

#### 5.4.2. Optimisation-Based

We propose a convex relaxation of the combinatorial search for $\max_{i\neq j}\langle c_{i},c_{j}\rangle$ in (1). To this end, recall the convex log-sum-exp approximation of the maximum: For any $x\in R^{n}$ and any temperature parameter t>0, we have

(20) $$\max_{i}x_{i}\leq \frac{1}{t}\log\left(\sum_{i=1}^{n}\exp\left(tx_{i}\right)\right)\leq \max_{i}x_{i}+\frac{\log n}{t}.$$

For greater temperatures, log-sum-exp function approaches the maximum. By carefully selecting a temperature scheduler, we can balance the need to update multiple candidate prototypes and the need to approximate the original problem in (1). Hence, we propose solving the following problem:

(21) $$\begin{array}{c} \min_{C}\frac{1}{t}\log\sum_{i\neq j}\exp\left(t\langle c_{i},c_{j}\rangle \right). \end{array}$$

### 5.5. Analysing Previous Schemes

We now evaluate the hyperspherical prototypes proposed in Mettes et al. [18], Kasarla et al. [19] in light of our characterisation of the optimal hyperspherical separation in Theorem 1.

In [18], the original problem (1) was relaxed by updating the nearest neighbour of *each* prototype by solving the following problem:

(22) $$\begin{array}{cc} \min_{c}\; & \frac{1}{N_{C}}\sum_{i=1}^{N_{C}}\max_{j}M_{i,j},\\ s.t.\; & M=C^{T}C-2I,\\ \; & ∥c_{i}∥=1. \end{array}$$

Since the diagonal of $C^{T}C$ is 1, the subtraction of 2I avoids self-selection. The authors argue that, by updating more prototypes with respect to each projected gradient descent step, the convergence speed is improved. On a high level, this relaxation has the same aim as our log-sum-exp relaxation: to trade off the need for faster convergence speeds by relaxing the original problem. However, the authors do not investigate whether the solution to the relaxed problem is close to the original problem.

In Kasarla et al. [19], the authors relax the original problem (1) by restricting the pairwise distance between prototypes to be constant through

(23) $$\begin{array}{cc} \min_{C}\; & a,\\ s.t.\; & \langle c_{i},c_{j}\rangle =a,\;\forall i\neq j. \end{array}$$

They give a closed-form solution to this problem through the recursive update rule:

(24) $$\begin{array}{cc} c_{1} & =\left[\begin{array}{cc} 1 & -1 \end{array}\right]\in R^{1\times 2}, \end{array}$$

(25) $$\begin{array}{cc} c_{k} & =\left[\begin{array}{cc} 1 & -\frac{1}{k}1^{T}\\ 0 & \sqrt{1-\frac{1}{k^{2}}}c_{k-1} \end{array}\right]\in R^{k\times (k+1)}, \end{array}$$

which they show yields separation 〈ci,cj〉=−1(NY−1) in dimension $n=N_{Y}-1$. While the authors do not show it, we notice that this separation agrees with the converse bound in Theorem 1, and it is therefore *optimal*. However, it is inflexible since the closed-form solution only gives prototypes in dimension $N_{Y}-1$.

### 5.6. Numerical Examples

**Figure 3:**
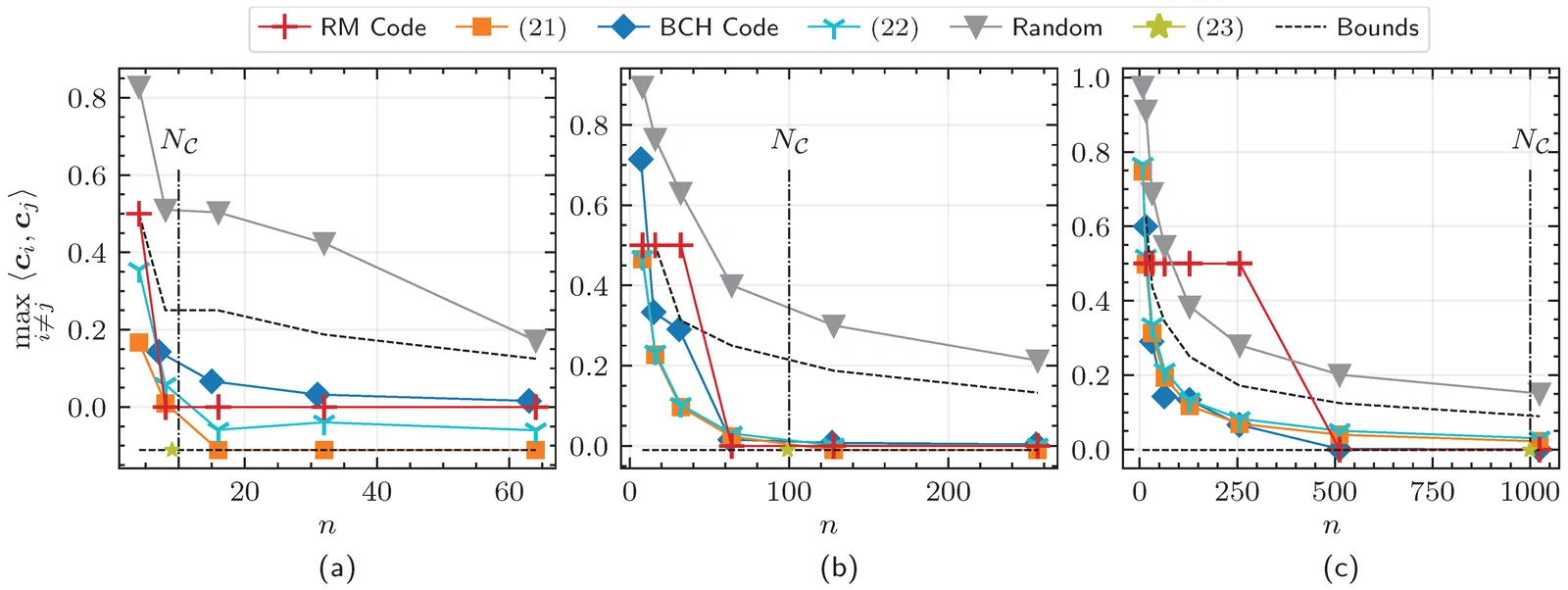
Worst-case prototype separation in various dimensions using various prototype generation schemes: (**a**) shows $N_{C}=10$, (**b**) shows $N_{C}=100$, and (**c**) shows $N_{C}=1000$ prototypes. Notice that the bounds from Theorem 1 are sharp in medium-scale to large-scale settings, where prototypes no worse than orthogonal prototypes are achievable, but minimum separation cannot be improved beyond near-orthogonality. Moreover, in the low-$N_{C}$ regime, optimisation-based prototypes give good worst-case separation, while in the large-$N_{C}$ regime, coding-theoretic prototypes give better worst-case separation.

We give results in varying latent space dimensions for $N_{C}\in \left\{10,100,1000\right\}$ classes (see Figure 3). For trainable prototypes, we follow the setup from Mettes et al. [18]: projected gradient descent with SGD (learning rate of 0.1 and momentum of 0.9 for 1000 epochs). In (21), we linearly increase the temperature from 1 to $N_{C}+1$.

We make three key observations. Firstly, the bounds from Theorem 1 are sharp in medium- to large-scale settings: No worse than orthogonal prototypes are achievable in low dimensions, and it is impossible to improve minimum separation beyond near-orthogonality. Note that the achievability (upper) bound says that good separation exists and can therefore be improved in low dimensions by noticing that both BCH and RM codes improve on it. Secondly, in the low-$N_{C}$ regime, optimisation-based methods perform well. Here, relaxations to the original problem (1) can yield optimal prototypes in large enough dimensions. We also see that the prototypes proposed by Mettes et al. [18] are suboptimal since they do not achieve the converse (lower) bound. Finally, in the large-$N_{C}$ regime, coding-theoretic prototypes work well. In this regime, good relaxations to (1) seem to be difficult, possibly due to the large number of pairwise distances.

Finally, we plot the distribution of inner products in Appendix C and make some qualitative interpretations of the different schemes.

## 6. Results and Discussion

In this section, we present numerical examples via CIFAR-100 and ImageNet, which show that (near-)optimal separation is neither necessary nor sufficient for downstream performance, and we reveal the importance of optimising prototype labelling.

### 6.1. Experimental Setup for Classification

We use the following experimental setup for classification tasks, which largely uses standard hyperparameters. On CIFAR-100, we use a ResNet-34 backbone with standard hyperparameters (SGD with learning rate of 0.2, momentum of 0.9, weight decay of $5\cdot 10^{-4}$ and batch size of 512 over 200 epochs, with a cosine learning rate scheduler). Data augmentation is also standard (random 32×32 crops with padding 4, random horizontal flips with probability 12, and random rotations of up to 15°). We carry out training using loss (3) with AutoShuffle sparsity penalties, $\lambda =10^{-3}$, and softmax temperature of τ=1 if nothing else is stated. Additionally, we randomly extract a validation set comprising 10% of the training set to pick hyperparameters. This increases randomness in the final performance but ensures that the test-set performance is an unbiased estimator of performance on unseen data.

For the sparsity penalty, we do not use weight decay, since minimising the L2-norm of the matrix $Q_{Y|C}$ makes it more dense. For this parameter, we use a linearly increasing learning rate scheduler, which increases the learning rate from 0.01 to 0.1 for 100 epochs and thereafter is held fixed. Intuitively, at the start of training, latent representations are mostly random, so we do not want to decide on relabelling immediately. Instead, we start by extracting features and then learn the relabelling of the prototypes.

For completeness, we also provide baselines of one-hot encoding and the prototypes from (22) and (23). Both one-hot encoding and (23) are agnostic to relabelling since the pairwise distance between any two prototypes is constant, and therefore, we do not employ trainable relabelling for these. Moreover, the latent representations for one-hot encoding are, as usual, not restricted to the hypersphere.

On ImageNet, we use a ResNet-50 backbone with the same hyperparameters, with the exception that we use different data augmentation methods (random resized crop extracting 224×224 images and random horizontal flips with a probability of 12).

### 6.2. Classification on CIFAR-100

**Figure 4:**
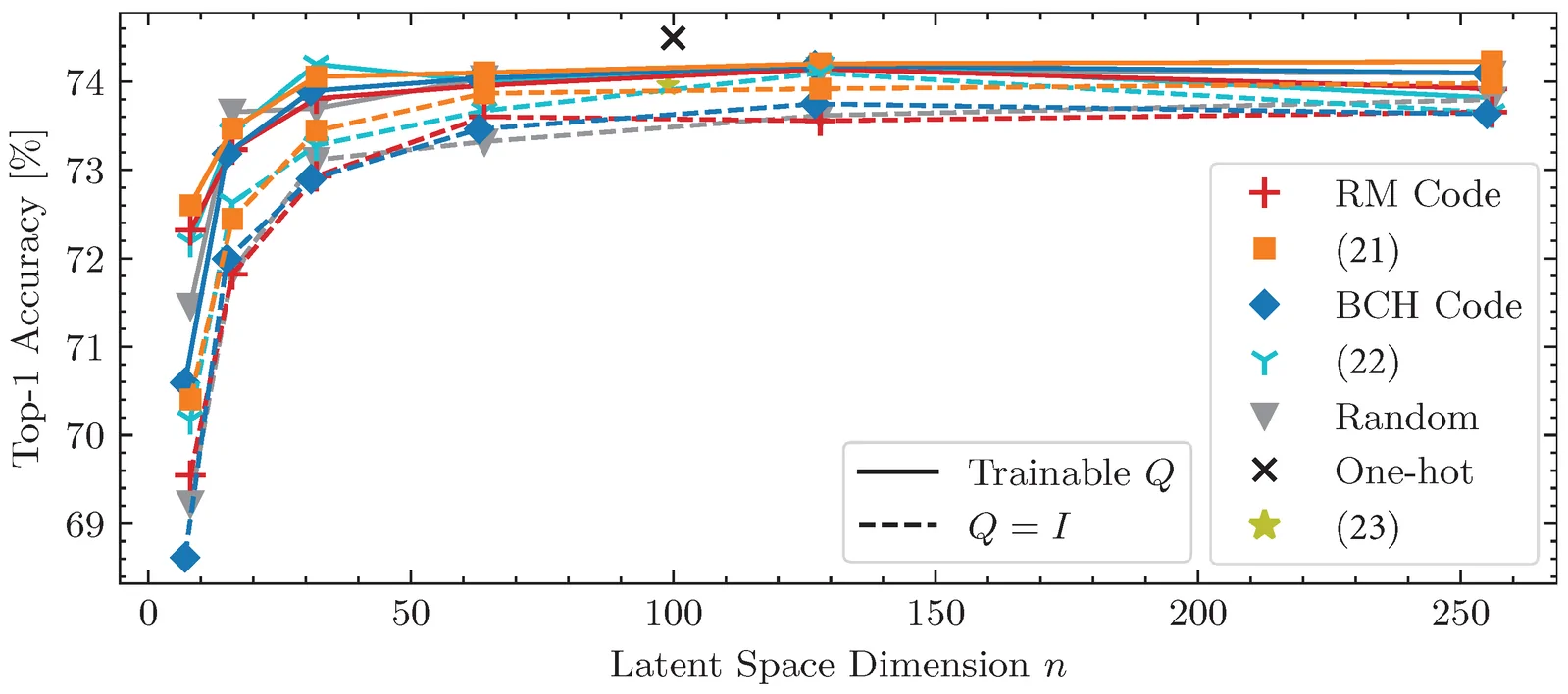
Accuracy on CIFAR-100 in various dimensions using various prototype generation schemes. We are able to maintain accuracy in dimensions as low as n=16 across prototypes with varying separation. We also find that trainable relabelling almost always helps: This holds for almost all prototype separations and dimensions. Moreover, good relabelling can, in many cases, compensate for poor prototype separation (for example, for random prototypes), which shows that good separation is not necessary for good performance. Finally, we notice that several schemes with similar separation exhibit different performance, which shows that good separation is not sufficient for good performance.

We investigate classification with varying prototypes and latent space dimensions (see Figure 4). The results are averaged over five runs to reduce variance. The results show that we are able to maintain accuracy in dimensions as low as n=16 with almost no performance loss and with only a small performance loss in dimension n=8.

Our first main conclusion is that there are consistent improvements with trainable relabelling. This observation holds across almost all prototypes and in almost all dimensions, and it highlights the importance of aligning the latent space geometry to the data by adapting prototype labelling.

Our second main conclusion is that good separation is not necessary to reach good performance. This is particularly clear in the case of random prototypes. These have not been improved beyond random initialisation, and as shown in Figure 3, they have poor separation. Despite this, the performance is often similar to, or even exceeding, the performance of prototypes with provably near-optimal separation.

Our third main conclusion is that good separation is not sufficient to guarantee good performance. RM code-based prototypes are provably no worse than orthogonal prototypes in dimension 64, but their performance is still worse than other near-optimal schemes. For RM code-based prototypes, their performance is relatively poor in higher dimensions and across runs, showing that good separation is not sufficient to explain performance.

Our final main conclusion is that good latent space geometry is particularly important in low dimensions where there are fewer degrees of freedom to preserve input data structure. In low dimensions, we need more prototypes per dimension, which reduces the degrees of freedom. On the other hand, in high dimensions, it is easy to create mutually orthogonal prototypes where labelling is less important. In the extreme case where pairwise separation is constant, labelling does not matter at all: All relabelling instances result in equivalent latent space geometries. This observation leads us to a reinterpretation of the benefit of high-dimensional latent spaces: Learning representations in high dimensions is a way to avoid the need to consider geometry. Since it is easy to create mutually orthogonal features in high dimensions, there are enough degrees of freedom to preserve input data structures.

### 6.3. Scaling up to ImageNet

**Figure 5:**
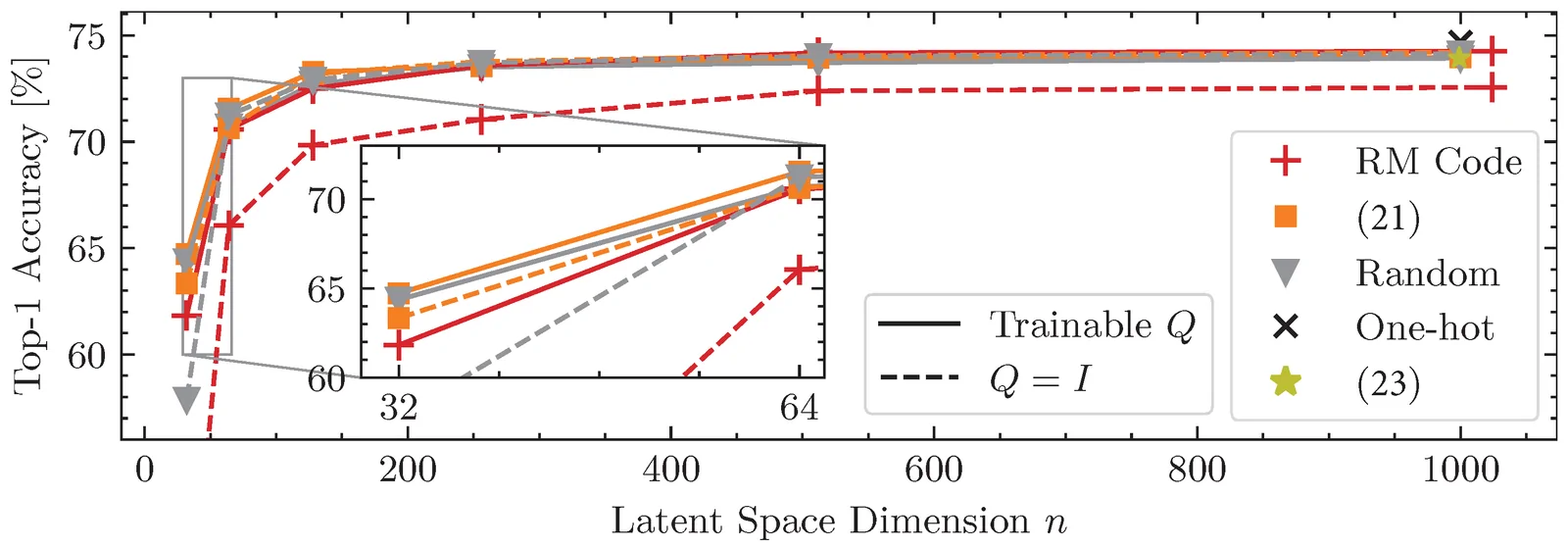
Accuracy on ImageNet in various dimensions using RM code, log-sum-exp, and random prototypes with and without trainable relabelling. We see that improving worst-case separation need not improve performance, and trainable relabelling can compensate for poor performance. This seems particularly important in low dimensions.

We scale up our experiment to ImageNet in Figure 5. Due to computational limitations, we focus on RM code, log-sum-exp, and random code-based prototypes with and without trainable relabelling: RM code and log-sum-exp-based prototypes are near-optimal in terms of separation, and random prototypes serve as a baseline.

In part, the results on ImageNet confirm our observations on CIFAR-100. We see that improving worst-case separation need not improve performance and that good relabelling can compensate for poor separation. This seems particularly important in low dimensions where we have fewer degrees of freedom to preserve input data structures.

However, the performance increase in high dimensions due to relabelling is not as significant on ImageNet as on CIFAR-100. This discrepancy is interesting, and we find two potential causes: Either latent space geometry becomes less important as the number of features to learn increases, or we are not able to find good relabelling. We find the former cause unlikely, since the more classes and features we have, the more important it should be that the latent space’s geometry is suitable. On the other hand, it is likely that we did not find good relabelling. The number of possible relabellings increases as $N_{Y}!$, making it much harder to find good relabelling in larger problems.

## 7. Conclusions

We summarise our contributions as follows: Firstly, we introduce a theoretically motivated analysis framework which enables us to separate the effects of cluster separation and labelling. Due to invariances in information-theoretic loss functions, these losses do not optimise geometry. That is, if prototypes with good separation but fixed labelling induce a detrimental geometry, then these loss functions will maximize information for this geometry but fail at adapting to the data structure. Secondly, we give a full characterisation of the best possible feature separation on the hypersphere. We show that prototypes no worse than orthogonal features are achievable in low dimension and that this phenomenon is near-optimal. Finally, we show empirically that inducing poor separation can be compensated if one allows for additional flexibility to preserve input data structure. This results in equally good average linear class separability. Our results also highlight the utility of high-dimensional latent spaces. The ratio of features to latent space dimensions is important; this is a form of “rate,” cf. Section 5.1. In particular, high dimensions (or “low rate”) are a way to avoid troublesome latent space geometry: there are enough degrees of freedom to preserve input data structure by default.

To conclude, we now discuss limitations and future work. In our supervised learning experiments, we have restricted our attention to end-to-end trainable schemes with backpropagation for simplicity. Given the importance of trainable relabelling to preserve input data structures, other schemes should be investigated. We leave this for future work. Promising alternatives to find good relabelling are the Hungarian algorithm [20], as well as sparse optimal transport methods [45,46,47].

Finally, we leave it to future work to propose new methods which advance benchmarks. 

## Figures and Tables

**Figure A1:**
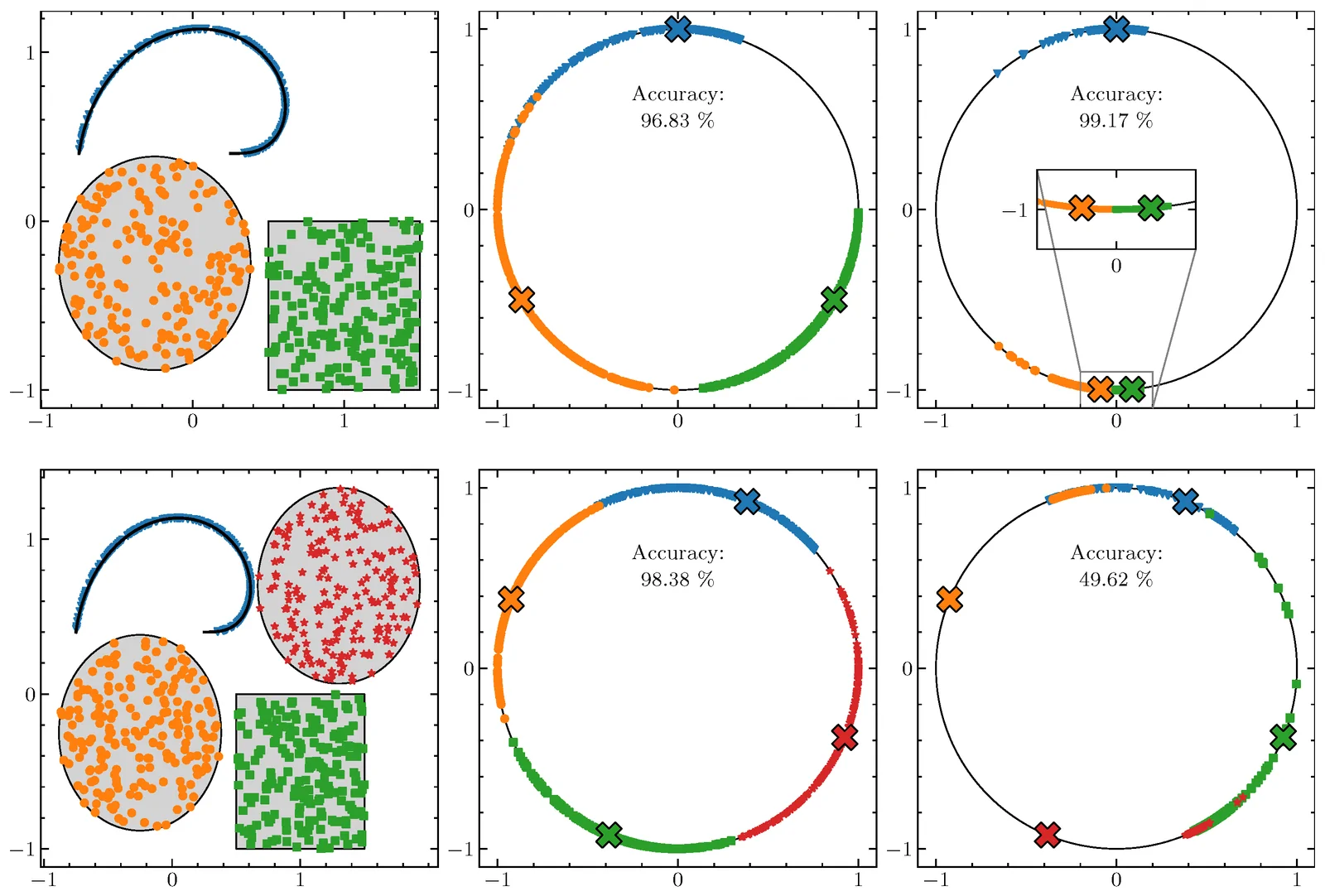
Example of separable toy data with a linear model. (**Top**) The effect of induced structure in the latent space. (**Left**) The test dataset. (**Centre** and **Right**) The latent representations for models trained with optimally and poorly induced separation, respectively. The poorly separated prototypes better preserve the structure of the input data and therefore achieve higher training accuracy with a linear model. (**Bottom**) The effect of preserving structure from the input data. (**Left**) The test dataset. (**Centre** and **Right**) show latent representations for two different prototype labellings. The labelling in (**Centre**) preserves the input geometry better than (**Right**) and achieves substantially better performance. The examples motivate a careful study of the importance of structure-inducing and structure-preserving inductive biases.

**Figure A2:**
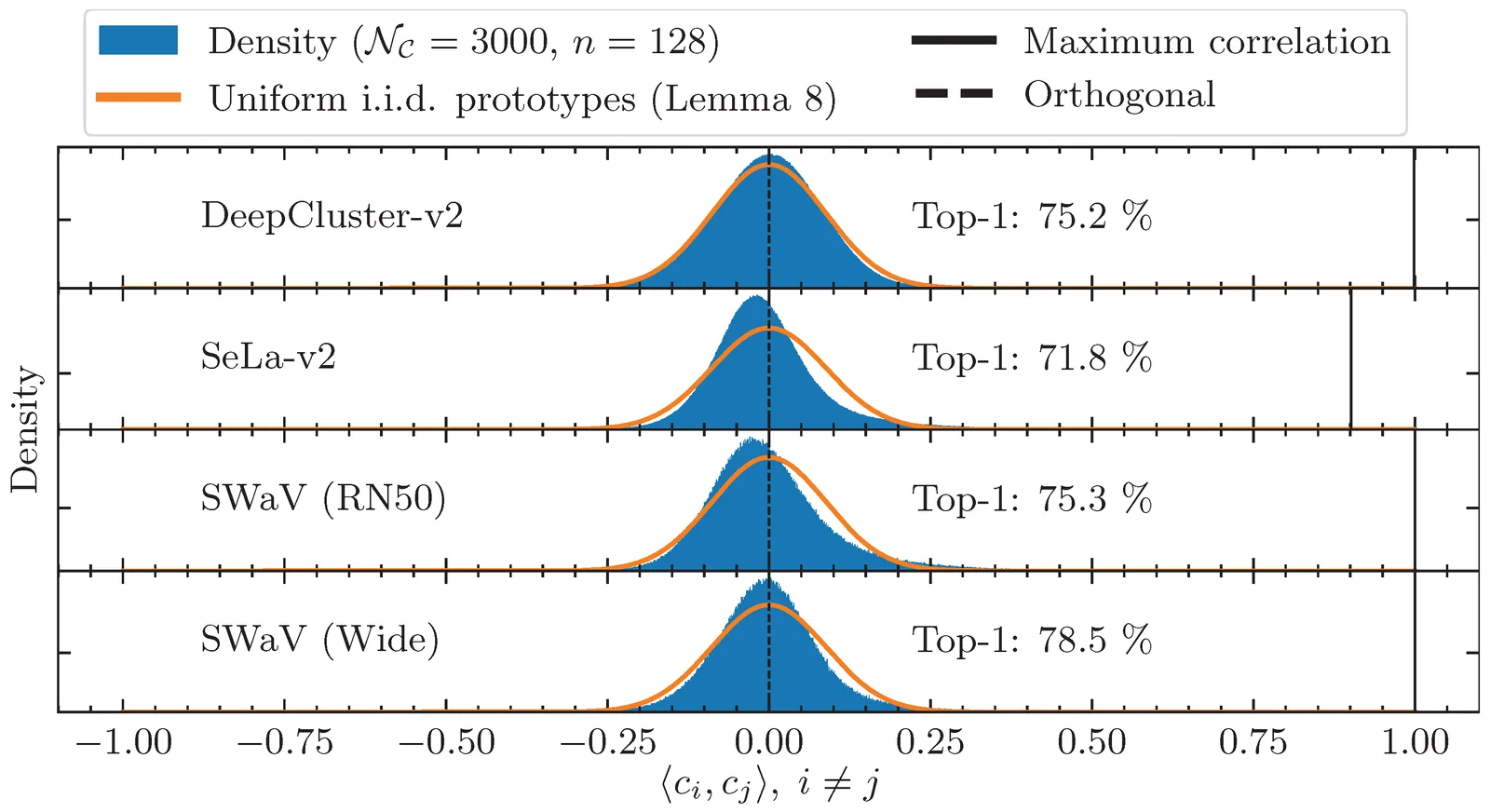
Hyperspherical prototype separation compared for pre-trained self-supervised models [8,48]. We compare separation histogram for prototypes generated with different methods compared to a baseline of the separation of untrained prototypes (i.e., drawn uniformly at random from the hypersphere). The dashed vertical lines denote orthogonality, and the solid vertical line shows the worst-case separation. Additionally, we provide the top-1 accuracy on ImageNet using linear classification on frozen features. We notice two things: The distributions do not differ substantially from those expected of prototypes drawn uniformly at random, and the downstream performance does not seem to correlate to either average separation or worst-case separation.

**Figure A3:**
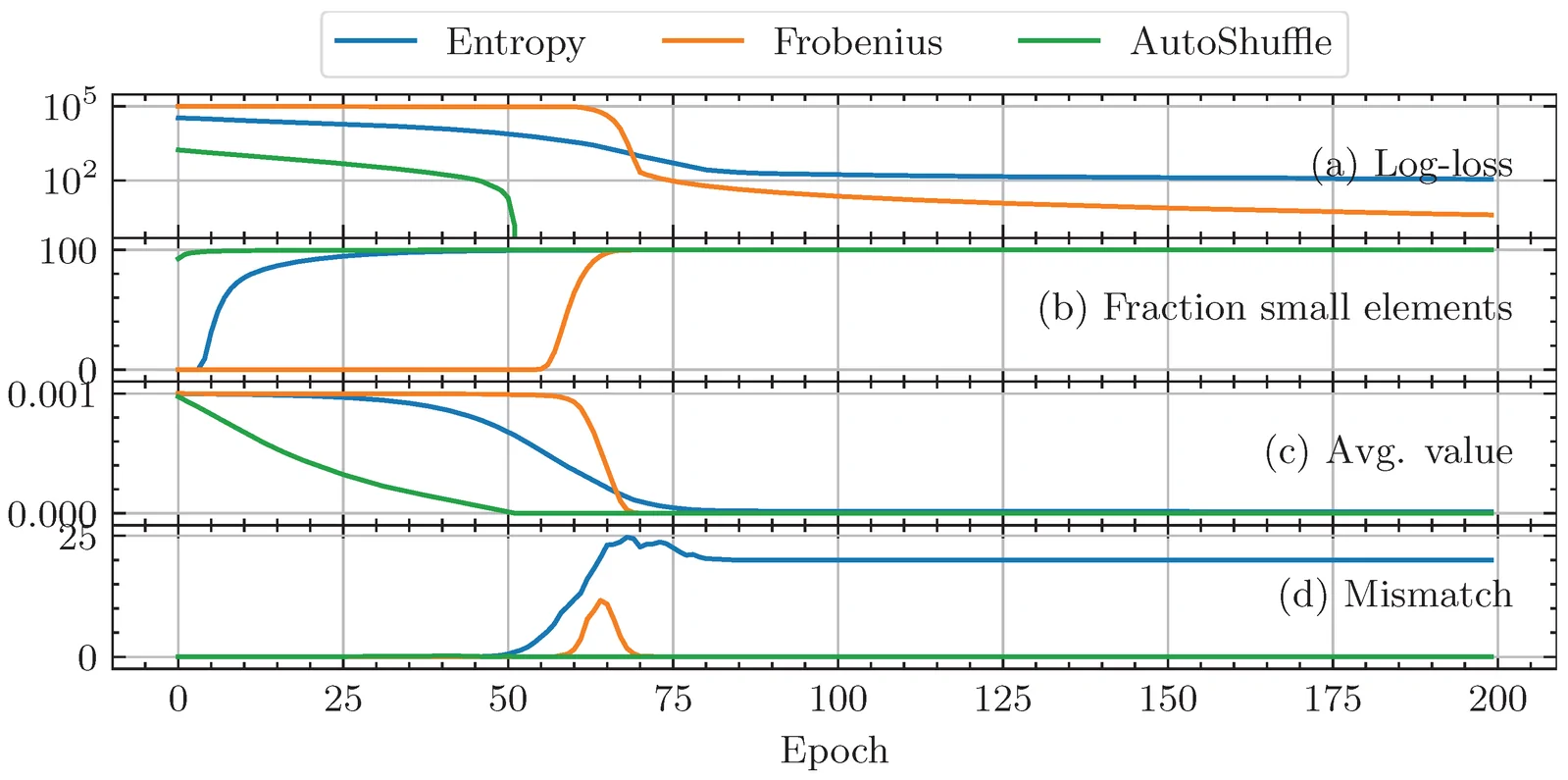
Compares sparsity and computational complexity for the Frobenius, entropy, and AutoShuffle losses. (**a**) How the loss decreases per epoch. (**b**,**c**) How well the losses measure sparsity: (**b**) the fraction values smaller than ε=0.1n−1 and (**c**) the average value of the n−1 smallest values per row. In (**d**), we plot the mismatch $∥M^{T}{1-1∥}_{1}$ between the column sum $M^{T}1$ and desired marginal 1. By construction, we always have M1=1. The results show that the AutoShuffle loss is relatively smooth with respect to sparsity, and the resulting matrix is nearly doubly stochastic. On the other hand, the Frobenius loss is not smooth with respect to sparsity. Furthermore, both Frobenius and entropy losses require many Sinkhorn iterations, which introduce significant computational overhead for large matrices.

**Figure A4:**
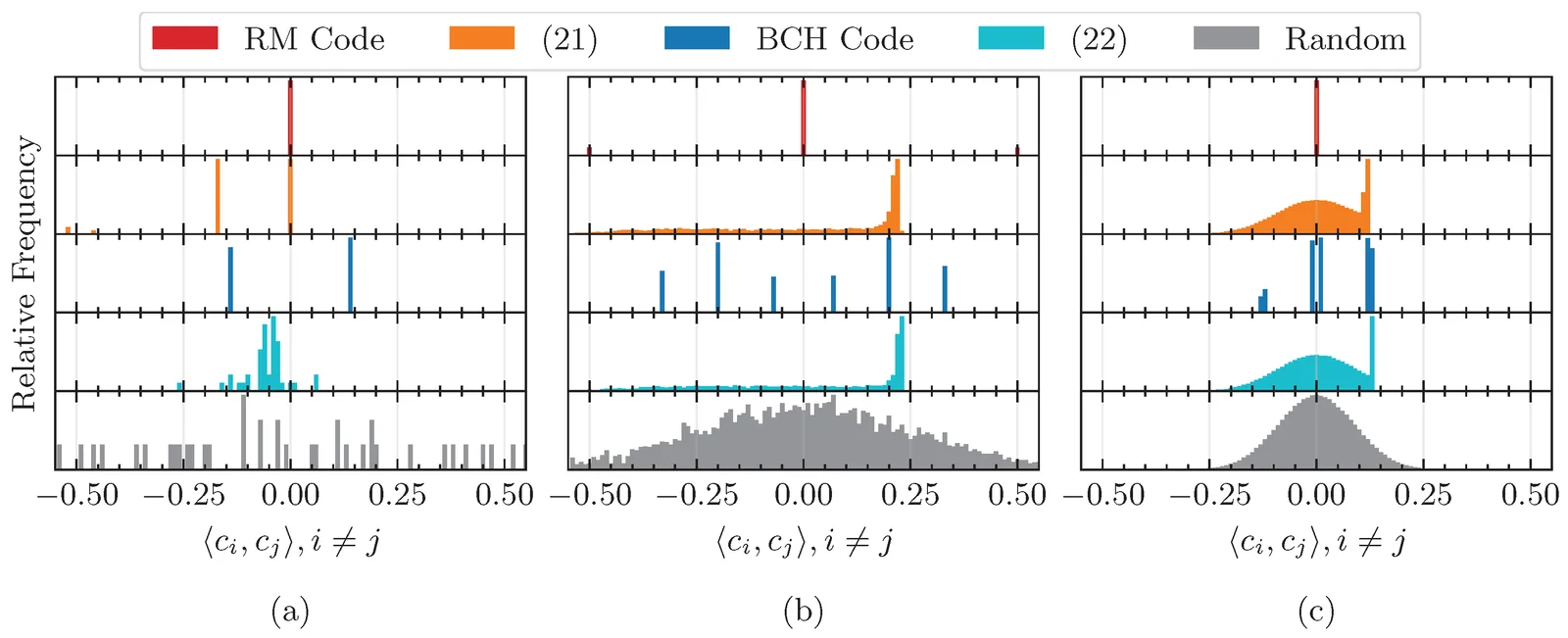
Prototype separation histograms for different schemes: (**a**) shows $N_{C}=10,n=8$; (**b**) shows $N_{C}=100,n=16$; and (**c**) shows $N_{C}=1000,n=128$. The plotting range is truncated for clarity. Note in (**b**,**c**) that there is non-zero density at similarity 0.5 for RM code-based prototypes.

## Data Availability

The datasets used in this paper are freely available to non-commercial research: CIFAR-100 is available at https://cave.cs.toronto.edu/kriz/cifar.html (accessed on 28 July 2026), and ImageNet is available at https://image-net.org/download.php (accessed on 28 July 2026).

## Appendix A. Motivating Examples

As a first motivating example, consider the toy example in Figure A1. The dataset (Figure A1, top left) consists of three equiprobable classes: (i) uniform distribution on a spiral arm parametrised by $\left(x_{1},x_{2}\right)=\left(\varphi \cos\varphi ,\varphi \sin\varphi \right)$; (ii) uniform distribution on a circle; and (iii) uniform distribution on a square. In Figure A1 (bottom left), we augment the dataset with an additional circle. In both cases, the data is separable and, additionally, nearly linearly separable. For each class, we place a *non-trainable class prototype* on the unit sphere. We train using a standard prototypical learning approach: For each sample (x,y), we want to map the normalised latent representations z=fW(x)∥fW(x)∥ as close to the corresponding prototype $c_{y}$ as possible. For a thorough background on prototypical learning, see Section 3.1.

Using training sets with 500 samples per class, we train a linear function, for which its output is later normalised. In other words, the mapping is linear, except for normalisation to unit length. Training is performed using standard hyperparameters (SGD with learning rate of 0.2, momentum of 0.9, weight decay of $5\cdot 10^{-4}$, and batch size of 512 over 200 epochs, with a cosine learning rate scheduler). For visualisation purposes, we evaluate and visualise the model using 200 samples per class.

In Figure A1 (top), we give an example of the effect of cluster separation on classification accuracy (linear separability): optimally separated clusters (top centre) can yield worse performance than adversarially chosen cluster separation (top right).

In Figure A1 (bottom), we give an example of the effect that cluster labelling can have: despite having the same cluster separation, two different labellings can yield substantially different performances.

This toy example shows that better cluster separation is not necessary or sufficient for good performance (see top) and that adversarial cluster geometry can severely degrade performance (see bottom). We emphasise that while perfect classification accuracy is possible, design choices may impact how easy they are to realise in practice.

As a second motivating example, we investigate pre-trained self-supervised models using DeepCluster-v2, SeLa-v2, and SwAV [8] in Figure A2. These methods all perform explicit clustering on the hypersphere using trainable cluster centres (called prototypes). We download and test pre-trained models from the official GitHub repository [48]. For SwAV, we compare the prototypes obtained on a ResNet-50 backbone, denoted by SwAV (RN50), and on the wider ResNet-50-w5, denoted by SwAV (Wide). These methods initialise the prototypes uniformly at random on the hypersphere and then update them using gradient descent. Therefore, for comparison, we also plot the distribution of the pairwise separation of random prototypes: In large dimensions, the distribution is closely approximated by 〈ci,cj〉∼N(0,1n). This is derived in Lemma A1 below.

We make two observations from the comparisons: Firstly, the training procedure does not seem to change prototype separation significantly. Moreover, the worst-case separation (solid line) is often around cosine similarity 1, meaning that some prototypes are almost coinciding, and the average separation is around 0, indicating that the mean separation does not improve substantially over random prototypes. Secondly, there does not appear to be a strong connection between learned prototype separation and linear class separability. For instance, the best-performing model (SwAV (Wide)) has small worst-case separation; on the other hand, the worst-performing model (SeLa-v2) has the best worst-case separation and average separation, which is better than orthogonal.

Similarly to before, we conclude that separation does not necessarily imply linear separability. Moreover, we highlight that, with trainable prototypes, it is hard to distinguish the importance of cluster separation from other design choices in the training algorithm.

Finally, we discuss the distribution of the inner product of points drawn i.i.d. from a uniform distribution on the hypersphere. Such points are easy to construct: Draw a vector $V∼N(0,I_{n})$ according to a standard normal distribution. Then, V∥V∥2∼U(Sn−1) is uniform on the unit hypersphere in dimension *n*. The distribution of the inner product $\langle c_{i},c_{j}\rangle$ can be derived in closed form.

**Lemma** **A1.**
*Let $c_{i}$ and $c_{j}$ be drawn i.i.d. and uniformly from the unit hypersphere in dimension n. Then, the inner product $\langle c_{i},c_{j}\rangle$ is related to the symmetric beta distribution through*

(A1)
$$\frac{1+\langle c_{i},c_{j}\rangle }{2}∼\beta \left(\frac{n-1}{2},\frac{n-1}{2}\right).$$

**Proof.** Since the uniform distribution on $S^{n-1}$ is rotationally symmetric, we may, without loss of generality, rotate the frame of reference such that $X=e^{\left(1\right)}$. Therefore, *Z* is distributed as the first component of *Y*. Recall that a uniform random vector can be generated through Y=V∥V∥2, where each component follows V(i)∼N(0,1) i.i.d. In particular, then, we have Z2∼β(12,n−12) ([49], Sec. 22.2): that is, $f_{Z^{2}}\left(z\right)\propto z^{-1/2}{\left(1-z\right)}^{(n-1)/2-1}$. Now, recall the change in the variable formula: If U=g(V) and *g* is monotone, then $f_{U}\left(u\right)=f_{V}\left(g^{-1}\left(u\right)\right)\left|\frac{d}{du}g^{-1}\left(u\right)\right|$. The claim now follows from two applications of this, firstly going from $Z^{2}\to Z$ with $g^{-1}\left(z\right)=z^{2}$, giving

(A2) $$f_{Z}\left(z\right)=f_{Z^{2}}\left(z^{2}\right)\left|2z\right|\propto {\left(1-z^{2}\right)}^{(n-1)/2-1}.$$

Finally, Z→Z+12 with $g^{-1}\left(z\right)=2z-1$ yields

(A3) f1+Z2(z)=2fZ(2z−1)∝(1−(2z−1)2)(n−1)/2−1∝z(n−1)/2−1(1−z)(n−1)/2−1,

which is a symmetric beta distribution with parameter n−12. □

Note that for a large *n*, the symmetric beta distribution is closely approximated by a Gaussian distribution, resulting in the approximation 〈ci,cj〉∼N(0,1n).

## Appendix B. Evaluating Sparsity Penalties

We now study the three candidate sparsity penalties. Birkhoff’s theorem establishes strong connections between permutation matrices and doubly stochastic matrices (see, for example ([50], Ch. 8.7)). For our purposes, it is enough to recognise that relabelling is a maximally sparse doubly stochastic matrix and that doubly stochastic matrices can be found with Sinkhorn’s algorithm [32].

The computational and memory overhead of this approach is small if the number of Sinkhorn iterations are small. The memory overhead of the doubly stochastic matrix scales quadratically with the number of classes, but it is manageable up to moderate problem sizes. The Sinkhorn iterations consist of iteratively normalising over rows and columns. Each of these operations is parallelisable and efficient, and the main expense is to run many iterations.

### Appendix B.1. Theoretical Analysis

We now consider three sparsity penalties, Frobenius, entropy, and AutoShuffle, and show that all of these can produce sparse matrices. The following proposition shows that the Frobenius norm can be used as a sparsity penalty.

**Proposition** **A1.**
*If $M\in R^{n\times n}$ is a doubly stochastic matrix, then ${∥M∥}_{F}^{2}\leq n$ with equality if and only if M is a permutation matrix.*

**Proof.** Recall that if $M\in R^{n\times n}$ is doubly stochastic, then $M^{T}1=M1=1$. To show this claim, consider the Frobenius norm ${∥M∥}_{F}^{2}=\sum_{i}{∥m_{i}∥}_{2}^{2}$, where $m_{i}$ denotes either rows or columns of M. We therefore consider each term $∥m_{i}{∥}_{2}^{2}$ individually. We shall show that $∥m_{i}{∥}_{2}^{2}\leq 1$ with equality if and only if $m_{i}$ is a one-hot vector or, in other words, maximally sparse. Since the decomposition ${∥M∥}_{2}^{2}=\sum_{i}{∥m_{i}∥}_{2}^{2}$ can be over both rows and columns, we can then conclude that ${∥M∥}_{F}^{2}\leq n$ with equality if and only if M is a permutation matrix. Hence, what remains is to investigate $∥m_{i}{∥}_{2}^{2}$. First, recall that ${∥v∥}_{2}^{2}\leq {∥v∥}_{1}^{2}$ for any vector v. In our case, this implies $∥m_{i}{∥}_{2}^{2}\leq {∥m_{i}∥}_{1}^{2}=1$, where the last equality is due to M being doubly stochastic. Now, notice that if $m_{i}$ is one-hot, then $∥m_{i}{∥}_{2}^{2}=1$. Hence, what remains is to show that if $m_{i}$ is *not* one-hot, then $∥m_{i}{∥}_{2}^{2}<1$. To this end, assume that $m_{i}$ has k>1 non-zero elements. Without loss of generality, we can then write $m_{i}=\sum_{j=1}^{k}\delta_{j}e^{\left(j\right)}$, where $e^{\left(j\right)}$ is the *j*-th canonical basis vector, and $\delta_{j}>0$ sum to 1. By the strict convexity of the 2-norm, we then get $∥m_{i}{∥}_{2}^{2}=∥\sum_{j=1}^{k}\delta_{j}e^{\left(j\right)}{∥}_{2}^{2}<\sum_{j=1}^{k}\delta_{j}{∥e^{\left(j\right)}∥}_{2}^{2}=1$. Since k>1 is arbitrary, this concludes the proof. □

In a similar manner as the Frobenius penalty, we can apply the entropy-like penalty $\ell_{\mathrm{sparse}}\left(\cdot \right)=\sum_{ij}m_{ij}\log m_{ij}$. The same arguments as that in Proposition A1 yields this claim, since for a row or column $m_{i}$ of M, the entropy $\sum_{j}m_{ij}\log m_{ij}=0$ if and only if $M_{i}$ is one-hot ([34], Theorem 1.4(a)).

The AutoShuffle loss [33] is a Frobenius-like penalty, but the matrix only needs to have non-negative entries (not strictly positive entries) and only needs to be approximately doubly stochastic. In practice, this means that we only perform *one* instance of row and column normalisation. Instead of these hard constraints, additional loss terms are added to penalise matrices which are not doubly stochastic through (4). See [33] for a proof that this penalty is 0 if and only if the matrix is a permutation matrix.

### Appendix B.2. Numerical Experiments

A candidate sparsity penalty should be fast to compute and track the number of non-zero elements of the matrix well. To do this, we compare the loss functions in Figure A3 on four metrics: the log-loss (a), fraction of near-zero elements (b), the average matrix value for the n−1 smallest values (c), and the impact of the number of Sinkhorn iterations (d). In (b), we say that an element $m_{ij}$ is small if mij≤ε=0.1n−1. This construction allows the contributions from the n−1 smallest elements to be at most 0.1. Taken together, (b) and (c) give an indication of how well the loss tracks the 0-norm. Finally, in (d), we track how close the column sum is to 1 through $∥M^{T}{1-1∥}_{1}$: Since Sinkhorn iterations normalise over row last, this measures whether the number of iterations is sufficient.

For comparison, we note that, in many settings, relatively few Sinkhorn iterations (around 3–4) are sufficient to get an approximately doubly stochastic matrix (see, for example, [8,46]).

To investigate this, we consider a typical learning scenario on ImageNet: We use 1000×1000 matrices and train for 200 epochs, each of which consists of 2505 batches, which corresponds to an ImageNet batch size of around 512. We use an SGD optimiser with a momentum of 0.9 and a cosine learning rate scheduler, where we tune the learning rate of the different losses to achieve sparsity after roughly the same number of epochs. We initialise the elements of all matrices uniformly in [0,2]. For Frobenius and entropy losses, we run the Sinkhorn algorithm for eight iterations, which is twice as many iterations as commonly used [8,46], and we utilise a SoftPlus activation to ensure that all elements are strictly positive. For the AutoShuffle loss, we follow the algorithm in Lyu et al. [33]: After each gradient update step, ReLU and one Sinkhorn iteration is applied. See Figure A3 for a comparison of their behaviours.

The results show that the different losses do not necessarily track sparsity well. In particular, the Frobenius sparsity penalty exhibits step-like behaviour, both in terms of the number of small elements and the average size. In the entropy and AutoShuffle losses, the same problem is less pronounced.

In both the Frobenius and entropy losses, we want to impose a hard double stochasticity constraint. We see empirically that when the matrices change quickly, we need more Sinkhorn iterations to achieve double stochasticity. This observation has practical implications: The cost of running a Sinkhorn iteration scales with $n^{2}$, quadratically with matrix size. On the other hand, the soft double stochasticity constraint in AutoShuffle only requires one Sinkhorn iteration and is still close to doubly stochastic.

Based on these empirical observations, we use $\ell_{\mathrm{sparse}}=\ell_{\mathrm{AutoShuffle}}$ in our loss (3).

## Appendix C. Distribution of Prototype Separation

To provide qualitative insights, we plot histograms of the prototype separation in Figure A4 for special cases: (a) shows the small-scale setting $N_{C}=10,n=8$, (b) shows the medium-scale setting $N_{C}=100,n=16$, and (c) shows the large-scale setting $N_{C}=1000,n=128$.

Firstly, note that the optimisation-based prototypes move density from the right tails towards lower similarity, where log-sum-exp relaxation achieves slightly better worst-case separation. The coding-based methods have separation concentrated at a few different values. The location of these can be calculated from the weight distribution (or weight-enumerator) function of the corresponding binary code ([37], Chapter 2, §1). Moreover, note that in high dimensions, the distribution of random prototypes becomes more Gaussian.

Secondly, note that for few prototypes, the optimisation-based methods effectively move prototypes to larger separation. On the other hand, for a large number of prototypes, the number of pairwise distances grows, and coding-theoretic prototypes outperform the optimisation-based in terms of worst-case separation.
